# Effect of Acupuncture for Chronic Nonspecific Low Back Pain: Study Protocol for a Randomized Double-Blind, Placebo-Controlled Trial

**DOI:** 10.2196/78713

**Published:** 2026-04-29

**Authors:** Hiroyoshi Yajima, Miho Takayama, Tomoaki Takanashi, Tomohiro Tanaka, Morihiro Nasu, Takahiro Yamada, You Hiramatsu, Ted Kaptchuk, Judith Schlaeger, Jian Kong, Shigeki Tanaka, Nobuari Takakura

**Affiliations:** 1 Department of Acupuncture and Moxibustion, Tokyo Ariake University of Medical and Health Sciences, 2-9-1 Ariake, Koto-ku, Tokyo, 135-0063, Japan, 81 3-6703-7000, 81 3-6703-7100; 2 Graduate School of Media Design, Keio University, Kanagawa, Japan; 3 Program in Placebo Studies, Beth Israel Deaconess Medical Center, Boston, MA, United States; 4 Department of Human Development Nursing Science, College of Nursing, University of Illinois Chicago, Chicago, IL, United States; 5 Department of Psychiatry, Massachusetts General Hospital, Boston, MA, United States

**Keywords:** acupuncture, double-blind needle, protocol for randomized double-blind placebo-controlled trial, chronic non-specific low back pain, surface electromyography (sEMG), objective outcome, self-report outcome

## Abstract

**Background:**

Chronic nonspecific low back pain (CNSLBP) is the most common chronic condition worldwide. Although recommended in several clinical guidelines, high-quality double-blind studies supporting the evidence for acupuncture remain limited.

**Objective:**

This study aims to test the efficacy of penetrating acupuncture for CNSLBP using double-blind needles that blind both the acupuncturist and the participant, which provides the most rigorous methodology for acupuncture trials to date.

**Methods:**

This study was a randomized double-blind (acupuncturist-patient) placebo-controlled trial conducted at the Tokyo Ariake University of Medical and Health Sciences Acupuncture Clinic. Seventy-five adult participants with CNSLBP were randomized to genuine penetrating, skin-touch placebo, and no-touch placebo needles that do not touch the skin. Consistent with prior studies and clinical practice, acupoints on the bladder meridian were mainly used. The study has 2 primary outcomes: an objective outcome and a subjective outcome. The primary objective outcome is the flexion-relaxation phenomenon, an index associated with low back pain, assessed using surface electromyography (sEMG) recordings of the bilateral lumbar erector spinae muscles and hamstrings. The primary subjective outcome is pain intensity measured using a visual analog scale (VAS) before and after the intervention.

**Results:**

The Ethics Committee approved the study protocol of Tokyo Ariake University of Medical and Health Sciences in April 2018 (approval number: 0246). This study started in July 2018 and was completed in March 2025.

**Conclusions:**

This trial used a double-blind design and objective outcome measures to rigorously evaluate the efficacy of acupuncture for CNSLBP.

## Introduction

Low back pain (LBP) is one of the most common chronic conditions and has a lifetime prevalence of approximately 50%‐84% worldwide [1-3]. The Japanese Ministry of Health reported that the rate of subjective symptoms (91.2 men and 113.3 women per 1000 population) makes LBP the most common chronic complaint in Japan [4]. The medical costs of work-related LBP in Japan were estimated to be 82.14 billion yen (746.72 million euro or US $1.027 billion) in 2011 [5].

LBP can be divided into 2 types: specific LBP caused by structural disorders of the spine, such as spinal stenosis and radiculopathy, or another internal organ causing referred pain; and nonspecific LBP with no apparent cause, which accounts for the majority (85%) of LBP cases [6]. Over 50% of patients with acute nonspecific LBP do not consult a health care provider. Most patients who consulted a health care provider still had pain after 3 months that developed into chronic nonspecific LBP (CNSLBP) [7]. Pathoanatomical, physical, neurophysiological, psychological, and social factors are associated with inducing and aggravating CNSLBP [8]. Patients with CNSLBP do not improve if these associated factors are not considered by their health care provider, which then results in patients receiving standardized, monotonic treatments [7].

Physiological studies using surface electromyography (sEMG) reported the absence of the flexion-relaxation phenomenon (FRP) [9-11] in patients with CNSLBP. FRP is an electrical silencing of the erector spinae muscles with full trunk flexion. The absence of FRP is attributed to abnormal tissue loading and pain that is mechanically provoked due to maladaptive movements as compensation for injury and impaired motor control [9,11,12]. The FRP is typically absent in patients with LBP, making it a clinically relevant physiological marker. As an objective neuromuscular indicator, FRP may be particularly valuable in placebo-controlled trials for detecting genuine treatment effects [12,13]. Therefore, evaluation of FRP is especially useful in placebo-controlled clinical trials of acupuncture, where sham acupuncture might have a greater effect on self-reported pain than a placebo pill [14,15].

The main treatment for CNSLBP is nonsteroidal anti-inflammatory drugs (NSAIDs). NSAIDs have shown small to moderate pain improvement as compared with a placebo pill in most systematic reviews [16]. However, the Agency for Healthcare Research and Quality indicated that NSAIDs administered for LBP are associated with gastrointestinal and cardiovascular adverse events [17] and adverse drug reactions such as gastric ulcer with bleeding and anemia, myocardial infarction, stroke, and acute kidney trauma [18]. If there is no improvement with NSAIDs, opioids are often prescribed [19], despite a lack of evidence that supports the efficacy of opioids for CNSLBP [20,21] as well as the risk of habituation [19].

Many clinical guidelines recommend acupuncture for CNSLBP [22,23]. Meanwhile, the use of acupuncture is controversial for the treatment of CNSLBP due to the lack of high-quality evidence to support its efficacy [23]. A 2005 Cochrane review reported that acupuncture is more effective than sham interventions as a short-term measure for treating CNSLBP [24]. In contrast, the National Institute for Health and Care Excellence in the United Kingdom does not recommend acupuncture for LBP with or without sciatica [25]. Recent systematic reviews have continued to report modest short-term benefits of acupuncture for chronic nonspecific low back pain; however, the overall certainty of evidence remains low to moderate due to methodological limitations, including inadequate blinding, physiologically active sham controls, and reliance on subjective outcomes [26].

Behind these inconsistent conclusions, there is a fundamental issue of acupuncture research, that is a suitable placebo control for acupuncture needling. Some studies used shallow needling or needling in the wrong points as a placebo control. However, these placebo controls are inappropriate because needles are inserted into the body, whether shallow, at the wrong points, or not [27,28]. To solve this issue, Streitberger et al [29], in 1998, developed a single-blind (patient masking) placebo needle that does not penetrate the skin. Many clinical studies have used the Streitberger needle to demonstrate the effectiveness of acupuncture. This placebo needle that presses the skin has been used in acupuncture studies as the best device to minimize patient bias because it has been considered almost impossible to double-blind both patients and acupuncturists [30-32]. In clinical trials that used these single-blind placebo needles [29,33], the efficacy of the genuine acupuncture group was not revealed compared to that of the placebo group [26,34]. Therefore, researchers have argued that results can be distorted using sham acupuncture, including the Streitberger needle, which is not physiologically inert [35]. In addition to the placebo not being physiologically inert, unblinded practitioners could have influenced how the patients responded [36], which introduces bias, strongly indicating the need for double-blinding in acupuncture research [34,37]. To solve these problems, our team developed a double-blind needle that could blind both patients and acupuncturists, which consists of 3 needle types, including a genuine needle and 2 types of placebo needles. The placebo needles include a skin-touch needle similar in structure to the conventional, physiologically active needle and a no-touch, physiologically inert needle [38-43]. Thus, it is imperative to conduct randomized placebo-controlled trials of acupuncture under double-blind conditions since these studies have not been conducted [30-32].

The primary aim of this study was to determine whether penetrating acupuncture produces greater improvements in FRP and pain intensity compared with both skin-touch placebo and no-touch inert placebo needles.

In summary, our double-blind acupuncture clinical trial for CNSLBP offers rigorous testing of a critical treatment for LBP that will advance acupuncture and placebo science. Our trial methodology addresses the following: (1) lack of practitioner blinding, (2) use of noninert placebo needles, and (3) lack of objective measures for the evaluation of LBP. The aims of our study are to (1) determine the efficacy of genuine penetrating acupuncture versus skin-touch placebo needles versus no-touch placebo needles and (2) assess the intervention effect using sEMG to record FRP, an objective measure of CNSLBP.

## Methods

### Study Design and Setting

**Figure 1.**
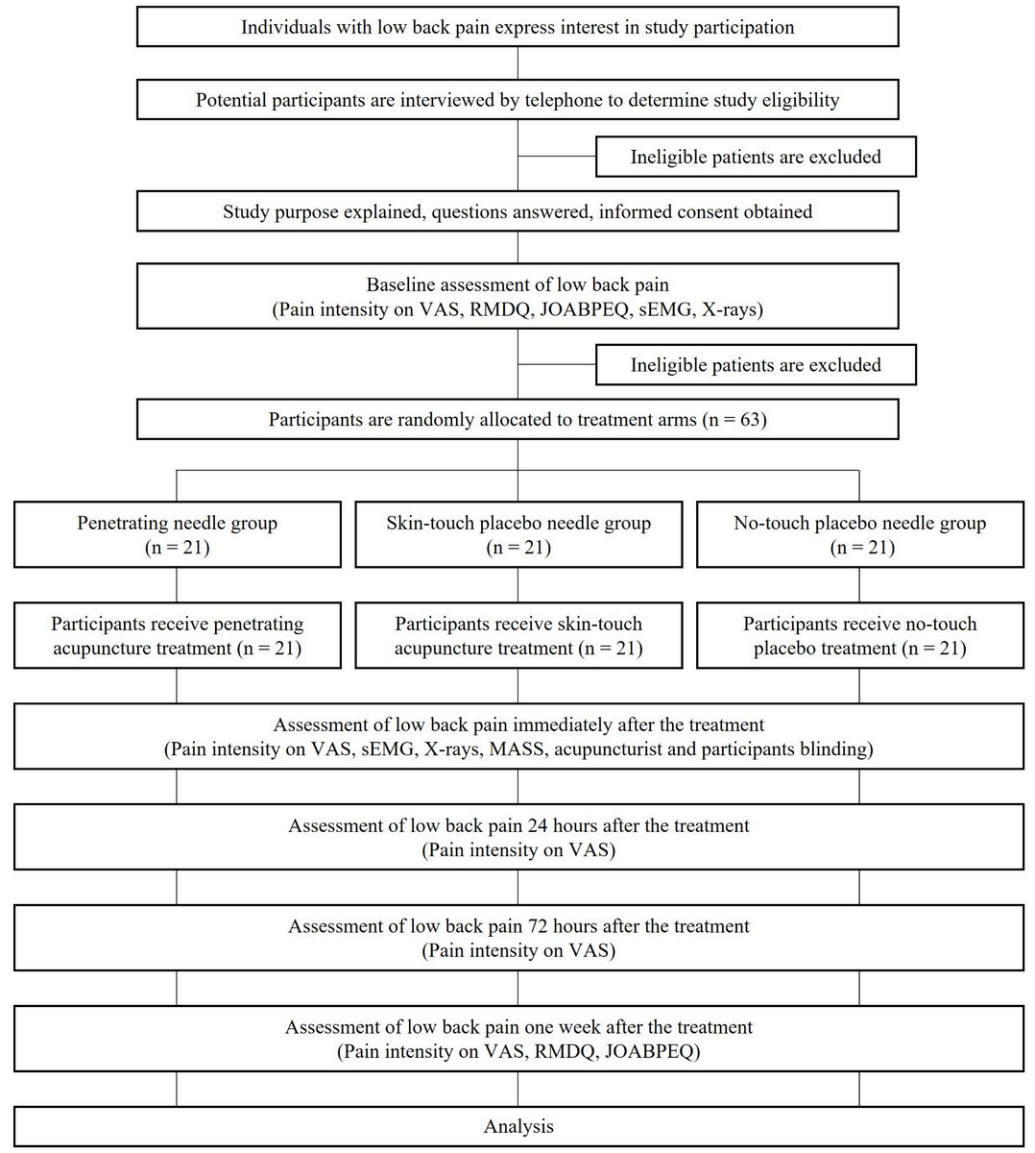
Study flow diagram. JOABPEQ: Japanese Orthopaedic Association Back Pain Evaluation Questionnaire; MASS: Massachusetts General Hospital Acupuncture Sensation Scale; RMDQ: Roland-Morris Disability Questionnaire; sEMG: surface electromyography; VAS: visual analogue scale.

This study is a single-center, randomized double-blind (acupuncturists and participants), placebo-controlled trial consisting of 3 treatment arms, including genuine penetrating needles (penetrating group), skin-touch needles (skin-touch group), and no-touch needles that do not touch the skin (no-touch group) [38-45]. This study was being conducted at Tokyo Ariake University of Medical and Health Sciences (TAU) Acupuncture Clinic and Medical Clinic, Japan. The study flow chart is shown in Figure 1.

### Sample Size

We calculated effect sizes and sample sizes using G*Power 3.1.9.6 (Heinrich-Heine-Universität, Düsseldorf). Results from a previous study of CNSLBP that determined the effects of high-velocity spinal manipulation on paraspinal muscle activity with sEMG found an effect size of 0.51 [46]. Based on this effect size, with a statistical power of 95% and an alpha of 5%, the sample size was calculated to be 63 participants (21 participants in each group). However, in our study, we conduct X-ray examinations at TAU Medical Clinic as a screening test for CNSLBP. Therefore, with a potential dropout rate of 20%, we recruited a total of 75 participants (25 participants in each group).

Because prior randomized trials evaluating acupuncture effects on FRP are lacking, the effect size was derived from the most physiologically comparable intervention study examining paraspinal muscle activity. The target power was set at 95% to reduce the probability of Type II error and to enhance the stability of the estimate in this mechanistic trial.

### Randomization

In total, 75 sets of double-blind needles were prepared and placed in separate bags numbered 1-75 (25 sets of 40 penetrating acupuncture needles, 25 sets of 40 skin-touch acupuncture needles, and 25 sets of 40 no-touch acupuncture needles). Seventy-five participants were randomly assigned 1:1:1 to the penetrating needle group, the skin-touch needle group, or the no-touch needle group by an independent investigator using a random numbers table that was generated by the RAND function (Microsoft Office Excel 2007).

### Data Management and Monitoring

The completed paper questionnaires are stored in a locked file cabinet for 10 years. Each participant was anonymized by coding without assigning any personal identification. Data from all outcomes were collected and stored in a database that restricts access by password.

The data manager, who was not directly associated with the study, oversees data monitoring activities throughout the study. Informed consent forms, data entry, completeness, and consistency were checked regularly to ensure data quality and protocol compliance.

### Inclusion Criteria and Exclusion Criteria

#### Inclusion Criteria

A physician from TAU Medical Clinic and a research assistant from TAU Acupuncture Clinic identify eligible participants based on the following criteria: (1) are aged 18-65 years, (2) have a diagnosis of CNSLBP, and (3) have other physical treatments such as physical therapy, massage, or take medications for CNSLBP one week before they are assigned to the treatment group and for the duration of the study.

Concomitant physical therapy, massage, or stable medication use is permitted for ethical and pragmatic reasons, reflecting real-world management of CNSLBP. Participants are instructed not to initiate new treatments during the study period. Randomization is expected to distribute co-interventions evenly across groups, thereby mitigating systematic confounding while preserving external validity.

#### Exclusion Criteria

Participants who have the following are excluded, including infections or tumors of the spine, lumbar herniation, rheumatism, lumbar vertebral compression fracture, scoliosis, osteoporosis, lateral curvature of the spine, central or peripheral neuropathy, sciatica, chronic neuropathic pain, circulatory disturbances of the lower limbs, hemorrhagic disorders, or taking anticoagulant medications, and are pregnant.

### Telephone Screening

The purpose and overview of the study were explained to potential participants over the telephone. They were then screened for eligibility. If participants met the inclusion criteria, they were given an appointment at the TAU Medical Clinic for a physical examination and x-rays to diagnose CNSLBP. Participants were also informed that they can withdraw from the study at any time.

### Baseline Assessment

On the first visit to TAU Acupuncture Clinic, the study’s objectives and methods were explained to participants using a written script. All questions are then answered, and study consents are signed. A physician at TAU Medical Clinic completed a physical examination, and an X-ray of the lumbosacral region of the frontal and sagittal planes was taken to confirm the morphological changes necessary to diagnose CNSLBP. Participants then completed all baseline questionnaires. The intensity of LBP was rated on a 100 mm visual analog scale (VAS) ranging from 0 (no pain) to 100 (most severe pain imaginable). The Roland Morris Disability Questionnaire (RMDQ) assessed physical disability due to LBP and contains 24 statements that described the discomfort that may occur with LBP. Participants were asked to check the responses that best describe their life on that day; the score was based on the total number of statements selected (0-24) [47]. The Japanese version of the RMDQ has been found to be reliable and valid for assessing patients with LBP [48]. The Japanese Orthopedic Association Back Pain Disease Questionnaire (JOABPEQ) assesses physical disability due to LBP and consists of 25 statements in 5 domains (back pain-related disorders, lumbar dysfunction, gait dysfunction, social life disorders, and psychological disorders) for the past 7 days. The JOABPEQ is scored with a range of 0-100 points; higher points indicate less physical disability [49]. The JOABPEQ has been found to be reliable and valid for assessing patients with LBP [50,51].

A research assistant then measured the distance between the floor and the middle fingertip during maximum lumbar forward bending, and the sEMG of the lower back was recorded. To obtain the sEMG, surface electrodes were attached to the bilateral lumbar erector spinae muscles 20 mm lateral to the L4 and L5 spinous processes [11,13,46,52] and on the hamstrings (midpoint on the line between the ischial tuberosity and the lateral epicondyle of the tibia) [53]. The sEMG was recorded using a Neuropack X1 (MEB-2306; Nihon Kohden Corporation) standing for 9 seconds, then maximum flexion for 3 seconds, and then maintaining the maximum flexion for 3 seconds, and finally a motion returning to the standing position for 3 seconds [11,13,46]. Also, the sEMG was recorded for 3 seconds in each of the following positions: back extension, right and left lateral bending, and right and left rotation of the waist.

### Intervention

#### Acupuncture Needles

**Figure 2.**
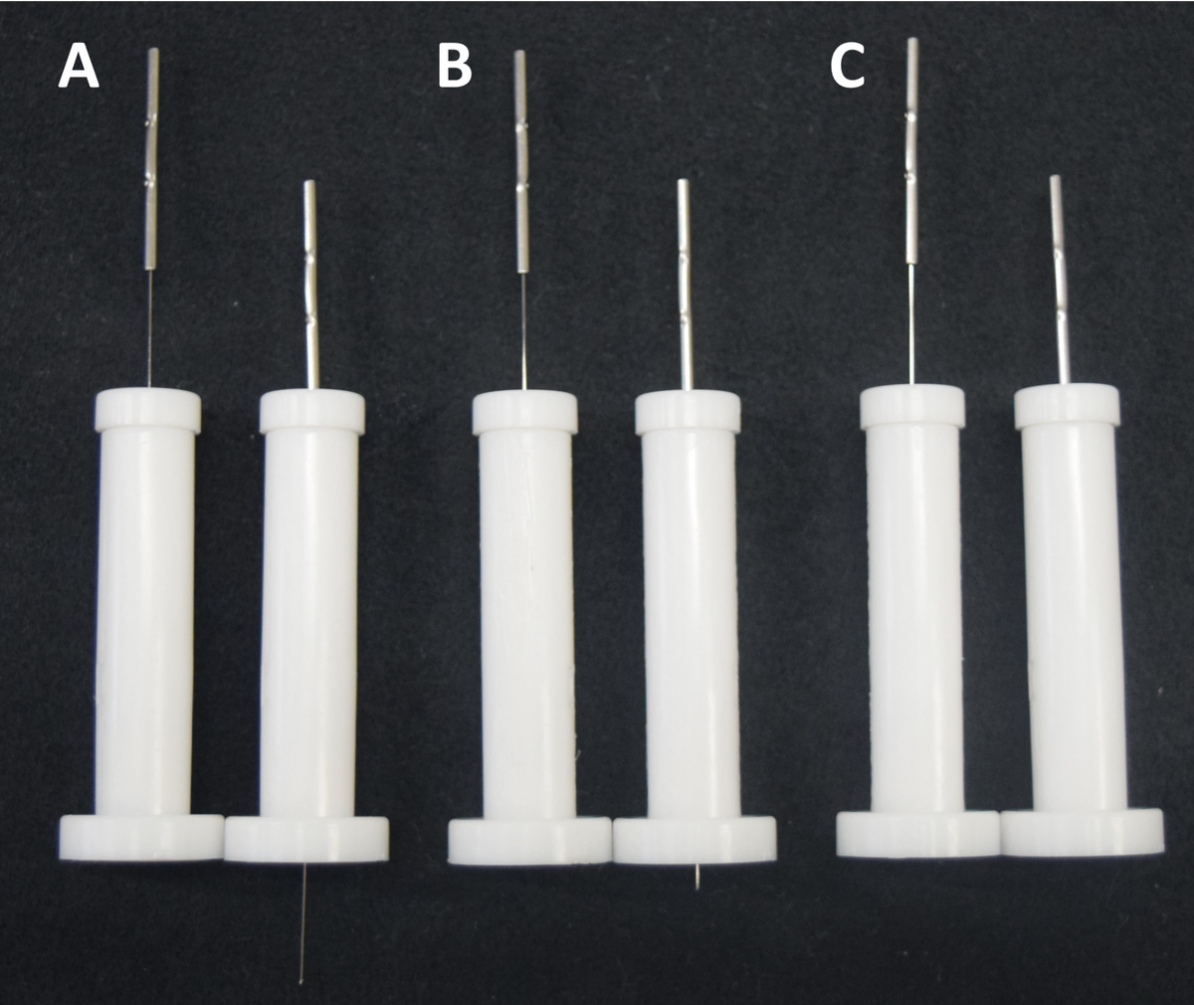
Double-blind needles before (on the left) and after insertion (on the right). (**A**) Penetrating needles inserted up to a depth of 20 mm, (**B**) skin-touch needles press on the skin to a depth of 2 mm, and (**C**) no-touch inert placebo needles. The penetrating needle in the photograph shows a depth of 10 mm.

Three types of double-blind needles (Confidence Co, Ltd; Figure 2) were used in this study: (1) genuine penetrating needles that pierced the skin and underlying tissue (Figure 2A; 5‐ to 20-mm depth depending on location and penetrating group), (2) skin-touch needles with a blunt tip that contacted the skin without penetration (Figure 2B; approximately 2 mm compression; skin-touch group), and (3) no-touch inert placebo needles that were designed so the tip did not contact the skin, providing a physiologically inert control condition (Figure 2C; no-touch group). From external appearance, all 3 needle types are indistinguishable. Therefore, it is not possible for participants and acupuncturists to make a judgment regarding the type of needle being used by how it looks. Also, there is silicone inside the inner guide tube that wraps around the needle and offsets the resistance to the needle insertion force [54]. This enables blinding of the participant and acupuncturist, as they cannot feel the needle type. The structure of the double-blind needles has been described in detail elsewhere [38-45,54-57]. The diameter of all 3 types of double-blind needles in this study was 0.20 mm. The penetrating needles were inserted to a depth of 5-20 mm (Table 1), depending on the anatomical location of where each needle was placed. The needles were sterilized using ethylene oxide gas prior to packaging.

**Table 1.** The acupoints’ location and insertion depth.

Acupoints	Location	Insertion depth
BL23 (Shenshu)	In the lumbar region, at the same level as the inferior border of the spinous process of the second lumbar vertebra, 1.5 cun lateral to the posterior median line.	20 mm
BL25 (Dachangshu)	In the lumbar region, at the same level as the inferior border of the spinous process of the fourth lumbar vertebra, 1.5 cun lateral to the posterior median line.	20 mm
BL26 (Guanyuanshu)	In the lumbar region, at the same level as the inferior border of the spinous process of the fifth lumbar vertebra, 1.5 cun lateral to the posterior median line.	20 mm
BL28 (Pangguanshu)	In the sacral region, at the same level as the second posterior sacral foramen, and 1.5 cun lateral to the median sacral crest.	20 mm
BL36 (Chengfu)	In the buttock region, at the midpoint of the gluteal fold.	20 mm
BL37 (Yinmen)	On the posterior aspect of the thigh, between the biceps femoris and the semitendinosus muscles, 6 cun inferior to the gluteal fold.	20 mm
BL40 (Weizhong)	On the posterior aspect of the knee, at the midpoint of the popliteal crease.	5 mm
BL52 (Zhishi)	In the lumbar region, at the same level as the inferior border of the spinous process of the second lumbar vertebra, 3 cun lateral to the posterior median line.	20 mm
BL54 (Zhibion)	In the buttock region, at the same level as the fourth posterior sacral foramen, 3 cun lateral to the median sacral crest.	10 mm
BL56 (Chengjin)	On the posterior aspect of the leg, between the 2 muscle bellies of the gastrocnemius muscle, 5 cun distal to the popliteal crease.	10 mm
BL57 (Chengshan)	On the posterior aspect of the leg, at the connecting point of the calcaneal tendon with the 2 muscle bellies of the gastrocnemius muscle.	10 mm
BL58 (Feiyang)	On the posterolateral aspect of the leg, between the inferior border of the lateral head of the gastrocnemius muscle and the calcaneal tendon, at the same level as 7 cun proximal to BL60.	5 mm
BL59 (Fuyang)	On the posterolateral aspect of the leg, between the fibula and the calcaneal tendon, at the same level as 3 cun proximal to BL60.	5 mm
BL60 (Kunlun)	On the posterolateral aspect of the ankle, in the depression between the prominence of the lateral malleolus and the calcaneal tendon.	5 mm
GB30 (Huantiao)	In the buttock region, at the junction of the lateral one-third and medial two-thirds of the line connecting the prominence of the greater trochanter with the sacral hiatus.	20 mm
GB31 (Fengshi)	On the lateral aspect of the thigh, in the depression posterior to the iliotibial band where the tip of the middle finger rests, when standing up with the arms hanging alongside the thigh.	10 mm
GB34 (Yanglingquan)	In the depression anterior and distal to the head of the fibula.	5 mm
Ex-B7 (Yaoyan)	In the lumbar region, at the same level as the inferior border of the spinous process of the fourth lumbar vertebra, 3.5 cun lateral to the posterior median line.	20 mm

#### Acupoints

BL23, BL25, BL26, BL28, BL36, BL37, BL40, BL52, BL54, BL56, BL57, BL58, BL59, BL60, GB30, GB31, GB34, and Ex-B7 (Table 1) were selected for use based on prior LBP studies [34,58-60].

#### Definition of De-Qi

Patient-perceived de-qi is suggested to be a sensation originating from deep tissues, rather than sharp pain from the lower border of the dermis as suggested by prior research [61,62]. Therefore, we defined it here as a painful sensation arising from deep tissues and asked patients whether they perceived it or not.

In addition, the de-qi perceived by the practitioner is termed “needle grasp,” suggested to be the “needle grasp sensation” felt by acupuncturists during insertion as they encounter various subcutaneous tissues [63]. However, practitioner-rated needle grasp sensation was not used as a procedural target to maintain blinding of the acupuncturists.

To standardize assessment and minimize interpretative variability, participants were asked whether they perceived a deep needling-related sensation. Additionally, the Massachusetts General Hospital Acupuncture Sensation Scale (MASS) was administered to quantitatively characterize sensory components of acupuncture needle insertion [64,65].

### Outcomes

All observed data are presented as means (SDs) and medians.

#### Primary Outcomes

The study includes 2 co-primary outcomes:

1. The immediate posttreatment change in the FRP measured using sEMG.
2. The immediate posttreatment change in pain intensity measured using a 100-mm VAS.

#### Secondary Outcomes

Secondary outcomes include:

1. Pain intensity was measured by VAS at baseline, 24 hours, 72 hours, and one week after treatment.
2. Physical disability assessed using the RMDQ at baseline and one week after treatment.
3. Back pain–related quality of life assessed using the JOABPEQ at baseline and one week after treatment.
4. Acupuncture sensation was assessed with the MASS immediately after treatment.
5. Practitioner and participant guesses regarding needle authenticity immediately after treatment.

### Primary Objective Measurement

The sEMG was recorded from the bilateral lumbar erector spinae at L4 and L5 and on the hamstrings before and after acupuncture.

### Primary Self-Report Measurement

The intensity of LBP was evaluated on a 100 mm VAS ranging from 0 (no LBP) to 100 (the most severe LBP imaginable) at baseline, immediately posttreatment, 24 hours, 72 hours, and one week after treatment.

### Secondary Self-Report Measurement

The following validated questionnaires were used to assess physical disability and acupuncture-related sensations at the specified time points.

1. RMDQ: the RMDQ assesses physical disability and was completed at baseline and one week after.
2. JOABPEQ: the JOABPEQ assesses physical disability with social life and their psychological effects, and it was completed at baseline and one week after.
3. MASS: the MASS consists of 12 descriptors of sensations to qualify and quantify acupuncture sensations. Participants select up to 12 descriptors that describe the sensations they had during their acupuncture treatment and then rate the intensity of these sensations on a numeric rating scale (NRS) from 0 (none) to 10 (strongest imaginable). If participants felt other sensations in addition to the 12 descriptors, they wrote a description of the additional sensation and rated it on the NRS [64,65]. The MASS was completed right after the acupuncture treatment was complete.
4. Blinding assessment: the practitioner and patient guess at the needle’s authenticity. The participants guessed whether the needle type was “penetrating,” “skin-touch,” “no-touch,” or “cannot identify the needle type” right after the acupuncture treatment was complete. The acupuncturists were also asked to guess the needle type and rated their confidence in their guess.

### Adverse Events

Even though they are rare, the research assistant and the acupuncturists monitor the participants for the presence of adverse events, such as pneumothorax, external or internal bleeding, and hematomas. Serious adverse events occurring during the study will be treated at the TAU Medical Clinic and reported to the Ethics Committee of the TAU.

The skin-touch needle features a blunt tip, a structural insertion stop, and a mechanism to physically prevent penetration and further pressure [38-45,54-57]. Previous validation studies and microscopic evaluation confirm its inability to pierce the skin under standard use [44]. Any unintended penetration would be recorded as a protocol violation and analyzed transparently.

### Treatment Procedure

Before treatment, the acupuncturist read the following script to each participant, “You are going to be randomly assigned to one of three groups: the penetrating needle group, the skin-touch needle group, or the no-touch needle group. The penetrating needle group is the treatment using real acupuncture needles that penetrate the skin, the skin-touch needle group is the treatment using skin-touch acupuncture needles that touch the skin but do not penetrate, and the no-touch needle group is the treatment with no-touch acupuncture needles with tips that do not touch the skin.” All participants were given one free real acupuncture treatment upon the return of their surveys one week later. Individuals not meeting study inclusion criteria were also offered free acupuncture.

Next, the acupuncturist asked the participant to point out the most severe stiffness or pain in the lumbar, gluteal, and posterior thigh regions. Participants then lay supine on a treatment table.

Before each needle application, the acupuncturist massaged each acupoint with the fingertip of the index finger of the nondominant hand in a circular motion for 2 seconds. This insertion technique is a commonly practiced method in Japanese-style acupuncture. The assistant peeled off the adhesive paper covering the bottom of the pedestal of each needle device, covered the needle with an outer guide tube, and handed it to the acupuncturist. Then, the acupuncturist placed the needle device on the acupoint, which was held in place by the adhesive. The acupuncturist stabilized the needle device with the nondominant hand and tapped the top of the needle handle, which protruded from the outer guide tube, 5-7 times using the index finger of the dominant hand [66].

After needle tapping-in, the acupuncturist removed the outer guide tube and inserted the needle further to its targeted depth by rotating it clockwise and counterclockwise using an alternating twirling technique [38-45,54,66]. This process was repeated until all needles were inserted. The needles were retained for 15 minutes.

After 15 minutes, the acupuncturist withdrew each needle to its initial position using an alternating twirling technique to remove them [38-45,54,66]. The needle was then stored in an opaque envelope to prevent the group allocation from being revealed. After all needles were removed, the research assistant checked for bleeding from each needled insertion site and wiped the blood away with a cotton ball and/or applied pressure if needed. The acupuncturist again massaged the area where the needle was inserted.

Then, the acupuncturist assessed the participant for back pain. If the participant had no pain, the treatment was complete. If the participant still had pain, the hardest indurations and the most tender areas in the lumbar erector spinae, quadratus lumborum, gluteus medius, and biceps femoris were treated using a Sparrow Pecking technique (rapidly inserting and withdrawing the needle tip within a 2 mm amplitude) using another needle from the same set of needles randomly assigned to the participant [13].

After the acupuncture treatment was complete, participants rated the intensity of their LBP on a 100 mm VAS. Participants then completed the MASS. The participants then guessed whether the treatment was “penetrating,” “skin-touch,” “no-touch,” or “cannot identify the needle type.” They reported their confidence level in their guesses on a 100 mm VAS ranging from 0 (no confidence) to 100 (full confidence) [37-43,52,63]. The acupuncturists were also asked to guess the needle type and rated their confidence in their guess. The sEMG of the lower back was recorded using the same procedure as before the intervention. One week after treatment, participants brought the completed questionnaires (intensity of LBP on VAS, RMDQ, and JOABPEQ) back to the Acupuncture Clinic. At that time, a research assistant offered them a free acupuncture treatment with regular acupuncture needles.

### Recruitment

Seven licensed, experienced acupuncturists (4 are listed as authors: TT, MN, TY, and YH) with more than 5 years of experience each, at TAU Acupuncture Clinic, administered the acupuncture treatments. We enrolled 75 participants with CNSLBP. Participants were recruited by advertisements listed on TAU websites, flyers placed on TAU campus notice boards, and at public facilities (city offices, gymnasiums, and surrounding high-rise apartment buildings). The advertisements provided a brief introduction to the study, a description of the target population with eligibility criteria, and study contact information. Participant recruitment began in October 2018.

### Statistical Analysis

Baseline characteristics will be assessed using Fisher exact test, ANOVA, and the Levene test. To evaluate the primary and secondary outcomes, we will use a linear mixed-effects model to account for the variability between groups and over time. This model will include “treatment group,” “time,” and their interaction as fixed effects. To address the potential influence of practitioner-related differences, “practitioner ID” will be included as a random effect. For post-hoc analyses, the Bonferroni correction will be applied to adjust for multiple comparisons. For nonnormally distributed data, the Mann-Whitney *U* test, Friedman test, or Kruskal-Wallis test will be used as appropriate. Blinding success will be evaluated by comparing the proportion of correct guesses in each group against chance level (33.3%) using a chi-square goodness-of-fit test. In addition, Bang Blinding Index will be calculated for both participants and acupuncturists to quantify the degree of blinding [55,57]. A Blinding Index close to 0 indicates successful blinding, whereas values approaching ±1 indicate unblinding. To account for variability among acupuncturists, analyses will be conducted under an intention-to-treat framework. Missing outcome data will be handled using multiple imputation under the assumption of missing at random. All statistical analyses will be conducted using SPSS Statistics version 31 (IBM SPSS Statistics) and R (R Foundation for Statistical Computing). Statistical significance will be set at a 2-sided *P* value of <.05.

### Ethical Considerations

The Ethics Committee of the TAU (approval no. 246, date of approval: April 23, 2018) approved the study protocol. In addition, the study protocol followed the SPIRIT (Standard Protocol Items: Recommendations for Interventional Trials) guidelines (Checklist 1). This study has been registered with the University Hospital Medical Information Network (UMIN ID: 000043725). All participants with CNSLBP are required to understand and approve the purpose of the study and sign a written informed consent form. All participant data were anonymized using coded identifiers. Data were stored in password-protected databases and locked storage facilities for 10 years. Participants received one free acupuncture treatment after completion of follow-up questionnaires as compensation for participation.

## Results

Recruitment started in October 2018. Data collection was temporarily suspended from April 2020 to September 2022 due to COVID-19 restrictions that prohibited in-person clinical research activities. Recruitment resumed thereafter, and data collection was completed in March 2025. There were no significant deviations from the original trial registration.

## Discussion

### Principal Findings

This study evaluates the short-term specific efficacy of penetrating acupuncture using both objective (FRP) and subjective (pain intensity) measurements in patients with CNSLBP. To achieve this, we compared 3 interventions, including a penetrating needle, a skin-touch needle, and a no-touch placebo needle that is physiologically inert, to better understand the key component factor of acupuncture. We hypothesized that FRP would be restored alongside improvements in subjective pain in the penetrating acupuncture group compared to the skin-touch and the no-touch placebo groups. This study aims to provide a better understanding of the specific versus nonspecific therapeutic components of acupuncture.

CNSLBP lasting more than 3 months with no apparent cause represents a large portion of patients with LBP and imposes a significant economic burden on many countries [1,2,4]. No definitive effective treatment for CNSLBP has yet been established. Acupuncture has been recommended as an empirically effective treatment for LBP based on the efficacy of acupuncture reported in unblinded and single-blinded studies [22,23]; however, the scientific basis of these findings is weak. The results of these studies may be biased due to a lack of acupuncturist and/or participant blinding [26,34,35]. Blinding acupuncturists, which is the most challenging problem in acupuncture research, as well as blinding participants, has the potential to increase study rigor.

In studies of CNSLBP using sham acupuncture as a control, the immediate effect of genuine acupuncture was no better than sham acupuncture for pain reduction [26]. Studies using these shams underestimate the effectiveness of acupuncture [35] because these sham techniques are physiologically active [67,68]. In this study, we solve this problem by using no-touch placebo acupuncture needles that do not stimulate the skin. The results of this study will begin to provide definitive results regarding the efficacy of acupuncture and sham acupuncture for the treatment of CNSLBP.

This study also uses FRP, which is an index of LBP [11,13,46]. FRP is recorded using sEMG, an objective measure, that reduces the potential to introduce bias into the study. Testing the efficacy of acupuncture by combining changes in self-reported and objective indicators enhances the rigor of the study results. Additionally, the study examines the immediate physiological effects after a single acupuncture session. Although acupuncture in clinical settings usually involves multiple sessions, evaluating the immediate response offers valuable mechanistic insight into the specific physiological effects of needle penetration. Understanding these short-term responses is an important first step toward clarifying the biological mechanisms behind acupuncture treatment.

By combining a rigorous double-blind design with both objective physiological measures and subjective clinical outcomes, this study aims to clarify the specific contribution of needle penetration to acupuncture efficacy in patients with CNSLBP.

### Strengths

The strength of our study lies in its rigorous methodological design, which addresses several critical challenges in acupuncture research. By using a triple-arm, double-blind design—consisting of a penetrating needle, a skin-touch placebo, and a physiologically inactive placebo—we effectively controlled for nonspecific therapeutic effects. Furthermore, the inclusion of objective physiological indicators (FRP) alongside subjective measurements significantly enhances the clinical rigor and reliability of our findings.

### Limitations

Despite these strengths, several limitations should be noted. First, since the primary objective was to evaluate immediate posttreatment effects following a single intervention, our findings may not be extrapolated to the long-term clinical efficacy of repeated acupuncture sessions. However, identifying these immediate physiological changes is a crucial first step in elucidating the underlying mechanisms of acupuncture for CNSLBP. Second, the design of the double-blind needles prevented practitioners from perceiving de-qi, as the device is specifically engineered to blind the practitioner to the depth of insertion. While some traditional perspectives emphasize the importance of de-qi, its definitive relationship with specific acupuncture efficacy remains inconclusive and warrants further rigorous investigation [69].

### Conclusions

This study has several distinctive methodological features, as outlined below:

1. This study is the first randomized double-blind (acupuncturist-participants) placebo-controlled trial to test the short-term effects of acupuncture on CNSLBP.
2. This study uses a genuine penetrating needle and 2 placebo needles, a skin-touch needle and a no-touch needle that is physiologically inert, to better understand the key component of acupuncture.
3. This study evaluates the effects of acupuncture on CNSLBP using both self-report measurement (pain intensity) and an objective measurement (activation of the erector spinae muscles using surface electromyography).

## Supplementary material

10.2196/78713 Checklist 1 SPIRIT 2025 checklist of items to address in a randomized trial protocol.

## Abbreviations

CNSLBP: chronic nonspecific low back pain
FRP: flexion relaxation phenomenon
JOABPEQ: Japanese Orthopedic Association Back Pain Disease Questionnaire
LBP: low back pain
MASS: Massachusetts General Hospital Acupuncture Sensation Scale
NRS: numeric rating scale
NSAIDs: nonsteroidal anti-inflammatory drugs
RMDQ: Roland Morris Disability Questionnaire
sEMG: surface electromyography
SPIRIT: Standard Protocol Items: Recommendations for Interventional Trials
TAU: Tokyo Ariake University of Medical and Health Sciences
VAS: visual analog scale
